# Effects of mindfulness-based interventions on negative emotions in patients with hypertension: a systematic review and meta-analysis

**DOI:** 10.3389/fcvm.2026.1941304

**Published:** 2026-10-30

**Authors:** Jiawei Lin, Changqun Lian, Guiting Zhou, Jiantao Liu, Yanlang Hua, Peijian Liu

**Affiliations:** 1 Department of Cardiology, Shunde Hospital of Guangzhou University of Chinese Medicine, Foshan, China; 2 Guangzhou University of Chinese Medicine, Guangzhou, China

**Keywords:** anxiety, depression, hypertension, meta-analysis, mindfulness

## Abstract

**Background:**

Psychological distress is common in hypertension and may impair disease management. Mindfulness-based interventions have been proposed to improve emotional and health-related outcomes, but evidence in this population remains limited.

**Methods:**

This systematic review and meta-analysis followed PRISMA guidelines. PubMed, Embase, Web of Science, the Cochrane Library, and CNKI were searched up to December 31, 2025, for randomized controlled trials of mindfulness interventions in adults with hypertension. Continuous outcomes were pooled as standardized mean differences (SMDs) with 95% confidence intervals (CIs) using fixed- or random-effects models. Risk of bias was assessed with the Cochrane tool, and the certainty of evidence was evaluated using the GRADE approach.

**Results:**

Nine trials with 780 participants were included. Mindfulness interventions significantly reduced depressive symptoms (SMD = −1.19, 95% CI: −1.74 to −0.64) and anxiety (SMD = −1.43, 95% CI: −2.48 to −0.39). Perceived stress was also reduced (SMD = −0.53, 95% CI: −0.82 to −0.24). Systolic (SMD = −2.10, 95% CI: −3.81 to −0.38) and diastolic blood pressure (SMD = −1.55, 95% CI: −2.69 to −0.42) were modestly reduced. Risk of bias was generally low to moderate. According to GRADE, the certainty of evidence was moderate for depression, systolic and diastolic blood pressure, and low for anxiety, perceived stress, and quality of life.

**Conclusions:**

Mindfulness-based interventions improve psychological outcomes in hypertension and may modestly lower blood pressure, supporting their use as a complementary strategy in hypertension management.

**Systematic Review Registration:**

https://www.crd.york.ac.uk/PROSPERO/, PROSPERO CRD420261328666

## 1 Plain language summary

High blood pressure affects over one billion people worldwide. Many patients with high blood pressure also struggle with stress, anxiety, or depression. These emotional problems can make it harder to control blood pressure and reduce quality of life. Blood pressure medications work well, but they do not treat emotional distress.

We wanted to know whether mindfulness-based interventions, such as meditation, breathing exercises, and body awareness training, could help. We searched medical databases and found 9 high-quality studies that tested these programs in 780 adults with high blood pressure. We combined the results to see the overall effect.

Our analysis showed that mindfulness-based interventions help patients with hypertension by reducing anxiety and depression, lowering stress levels, improving quality of life, and modestly lowering blood pressure.

These findings suggest that mindfulness programs can be a useful addition to regular medical care. They are low-cost, drug-free, and can be done in community settings. Doctors may consider recommending them to patients who struggle with emotional distress. However, more studies are needed to understand how long the benefits last and which programs work best.

## 2 Introduction

Hypertension affects approximately 1.28 billion adults worldwide and remains a leading contributor to cardiovascular disease, stroke, chronic kidney disease, and premature mortality (1). Despite the availability of effective antihypertensive medications, global control rates remain unsatisfactory, with fewer than half of treated patients achieving target blood pressure levels (2). In addition to traditional risk factors such as aging, obesity, and unhealthy lifestyles, psychosocial stress and emotional disturbances have been increasingly recognized as important contributors to the development and progression of hypertension (3).

Negative emotional states, particularly anxiety, depression, and chronic stress, are highly prevalent among patients with hypertension (4). Epidemiological studies have shown that these psychological conditions are associated with increased sympathetic nervous system activity, dysregulation of the hypothalamic–pituitary–adrenal axis, endothelial dysfunction, and systemic inflammation, all of which may contribute to elevated blood pressure and increased cardiovascular risk (5). Moreover, emotional distress is closely related to poor medication adherence, unhealthy behaviors, sleep disturbances, and reduced self-management ability. These factors not only compromise blood pressure control but also substantially impair health-related quality of life (QOL), which has become an important patient-centered outcome in hypertension management (6).

Mindfulness, defined as purposeful and nonjudgmental attention to present-moment experiences, has gained increasing attention as a behavioral approach for stress reduction and emotional regulation (6). Structured mindfulness-based interventions (MBIs), such as mindfulness-based stress reduction and mindfulness-based cognitive therapy, typically integrate meditation practices, breathing awareness, body scanning, and gentle movement training (7). These interventions aim to enhance cognitive flexibility, reduce emotional reactivity, and improve adaptive coping. Neurobiological and clinical studies suggest that mindfulness practice may modulate autonomic balance, reduce stress-related physiological arousal, and improve psychological resilience (8). In patients with chronic diseases, MBIs have been associated with reductions in anxiety and depressive symptoms, improved sleep quality, and enhanced overall well-being.

In the context of hypertension, mindfulness interventions may be particularly beneficial by addressing both psychological and behavioral pathways (9). By reducing negative emotions and perceived stress, mindfulness may indirectly improve blood pressure control through better adherence, healthier lifestyle choices, and improved self-regulation (10). In addition, improvements in emotional well-being may translate into meaningful gains in QOL. However, clinical trials examining the effects of mindfulness-based interventions in hypertensive populations have reported heterogeneous results, and psychological outcomes have not been systematically synthesized (11).

Therefore, this systematic review and meta-analysis aimed to evaluate the effects of mindfulness-based interventions on negative emotional symptoms in patients with hypertension, providing evidence to support their role in comprehensive hypertension management.

## 3 Method

### 3.1 Study design and data resources

This systematic review and meta-analysis was conducted and reported in accordance with the Preferred Reporting Items for Systematic Reviews and Meta-Analyses (PRISMA) 2020 statement (12). The study protocol was registered with the International Prospective Register of Systematic Reviews (PROSPERO) under registration number CRD420261328666 (available at https://www.crd.york.ac.uk/PROSPERO/).

A systematic literature search was performed in PubMed, Embase, Web of Science, the Cochrane Library, and China National Knowledge Infrastructure CNKI from database inception to December 31, 2025. The search strategy included combinations of keywords related to hypertension, mindfulness-based interventions such as mindfulness, MBSR, MBCT, and meditation, as well as psychological outcomes including anxiety, depression, stress, and quality of life. Boolean operators AND and OR were used to combine the search terms. In addition, the reference lists of relevant studies were manually screened to identify any additional eligible articles. The full search strategies for each database are provided in Supplementary Table 1.

### 3.2 Eligibility criteria

#### 3.2.1 Inclusion criteria

Studies were included if they met the following criteria: 1) Participants were adults (≥18 years) diagnosed with hypertension. 2) The intervention involved mindfulness-based approaches, including mindfulness-based stress reduction, mindfulness-based cognitive therapy, or other structured mindfulness programs. 3) The control group received usual care, wait-list control, health education, or other non-mindfulness interventions. 4) At least one outcome related to negative emotions or health-related quality of life was reported. 5) The study design was a randomized controlled trial.

#### 3.2.2 Exclusion criteria

Studies were excluded if they met any of the following conditions: 1) Non-randomized studies, observational studies, reviews, case reports, conference abstracts, or study protocols. 2) Mixed populations without separate data for hypertensive patients. 3) Interventions not primarily based on mindfulness. 4) Insufficient or non-extractable outcome data.

### 3.3 Study selection and data extraction

All retrieved records were imported into reference management software, and duplicates were removed. Two reviewers independently screened the titles and abstracts and then assessed the full texts of potentially eligible studies. Data were extracted using a standardized form, including study characteristics, participant demographics, intervention details, control conditions, outcome measures, and follow-up duration. The methodological quality of included studies was independently evaluated using the Cochrane Risk of Bias tool, which assesses the domains of randomization, deviations from intended interventions, missing outcome data, outcome measurement, and selective reporting. Any disagreements during study selection, data extraction, or risk of bias assessment were resolved through discussion or consultation with a third reviewer.

### 3.4 Statistical analysis

Meta-analysis was performed using R 4.3.3 and R studio software. Continuous outcomes were pooled as standardized mean differences (SMDs) with 95% confidence intervals (CIs). Heterogeneity was assessed using the I^2^ statistic. A random-effects model was applied when I^2^ > 50%; otherwise, a fixed-effects model was used. Sensitivity analyses were conducted to assess result stability. The quality of evidence for each outcome was assessed using the GRADE approach.

## 4 Result

### 4.1 Characteristics of included studies

A total of 1,101 records were identified through database searching. After screening titles and abstracts and assessing the full texts for eligibility, eight randomized controlled trials with a combined sample of 519 participants were included in this review (Figure 1). Sample sizes of the individual studies ranged from 30 to 112 participants, and the mean age of participants varied from 28 to 63 years. All studies (9, 13–19) compared mindfulness-based interventions with control conditions such as usual care or health education, and reported outcomes related to negative emotions (Table 1 and Supplementary Table 2).

**Figure 1:**
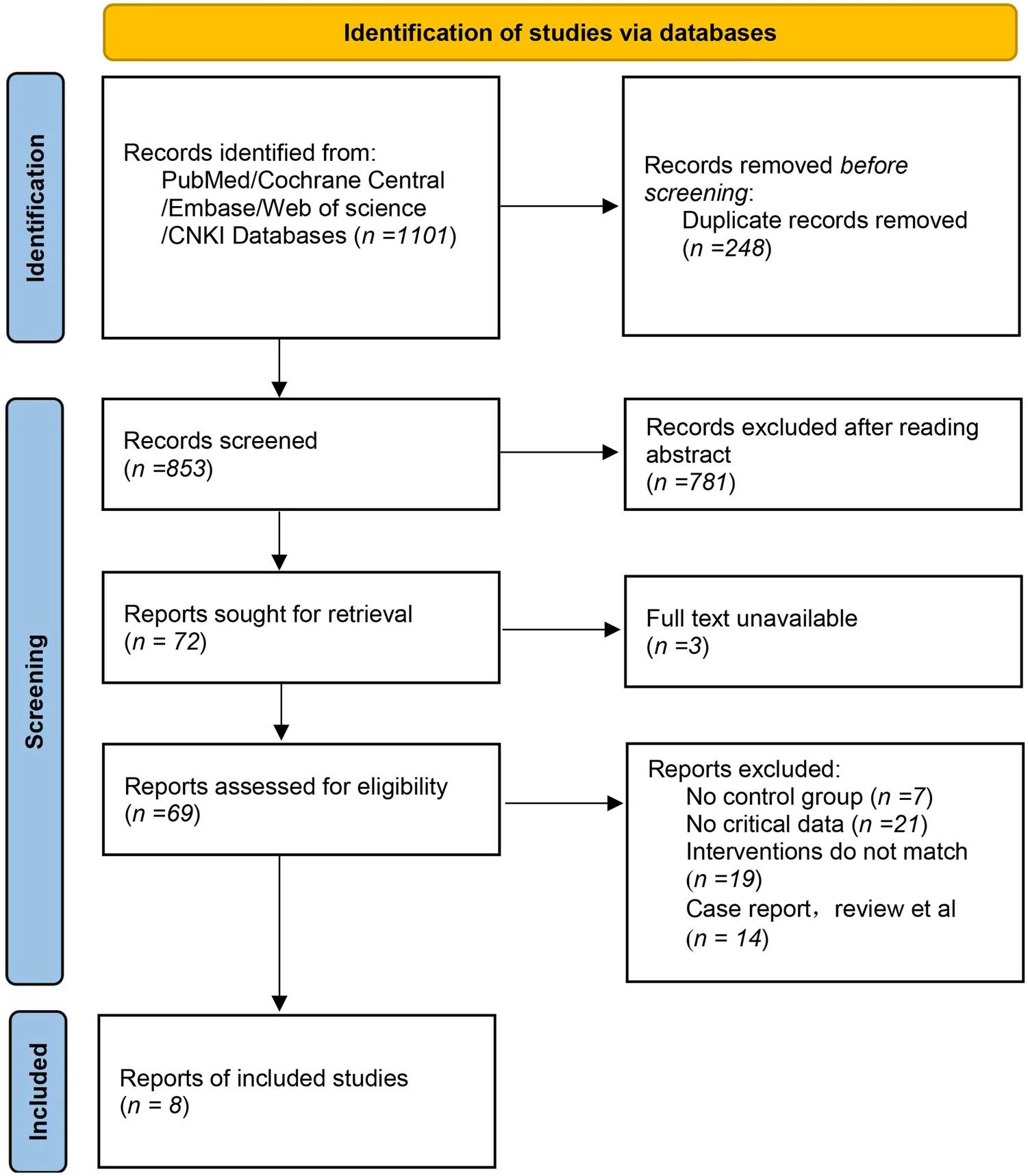
PRISMA 2020 flow diagram of the study selection process.

**Table 1:** Baseline characteristics.

First author	Publication year	Number of cases		Age (year)		Gender	
		Experimental group	Control group	Experimental group	Control group	Experimental group (Male/Female)	Control group (Male/Female)
Ahmadpanah (13)	2016	15	15	46.33 ± 2.67	46.69 ± 3.53	0/15	0/15
Chen (14)	2021	45	45	30 ± 1.26		0/45	0/45
Su (15)	2017	40	40	NR	NR	NR	NR
Polcari (9)	2022	14	22	61.7 ± 2.7	57 ± 3.4	8/6	11/11
Ponte (16)	2019	24	18	57.1 ± 5.4	55.7 ± 10.2	11/13	7/11
Wu (17)	2025	34	35	59.65 ± 10.35	63.17 ± 12.08	27/7	27/8
Yu (18)	2019	56	56	28.19 ± 4.57	29.45 ± 4.37	0/28	0/28
Zhang (19)	2024	30	30	60.02 ± 5.23	57.68 ± 7.71	8/22	14/16

### 4.2 Risk of bias assessment

The risk of bias was generally low to moderate across the included studies. Most studies reported adequate randomization, although allocation concealment and blinding were often unclear or at high risk. Outcome data were largely complete, and selective reporting was low in most trials. Some studies had additional concerns, such as small sample sizes or baseline imbalances. Overall, while the included trials provided useful evidence, the methodological limitations should be considered when interpreting the results (Figure 2).

**Figure 2:**
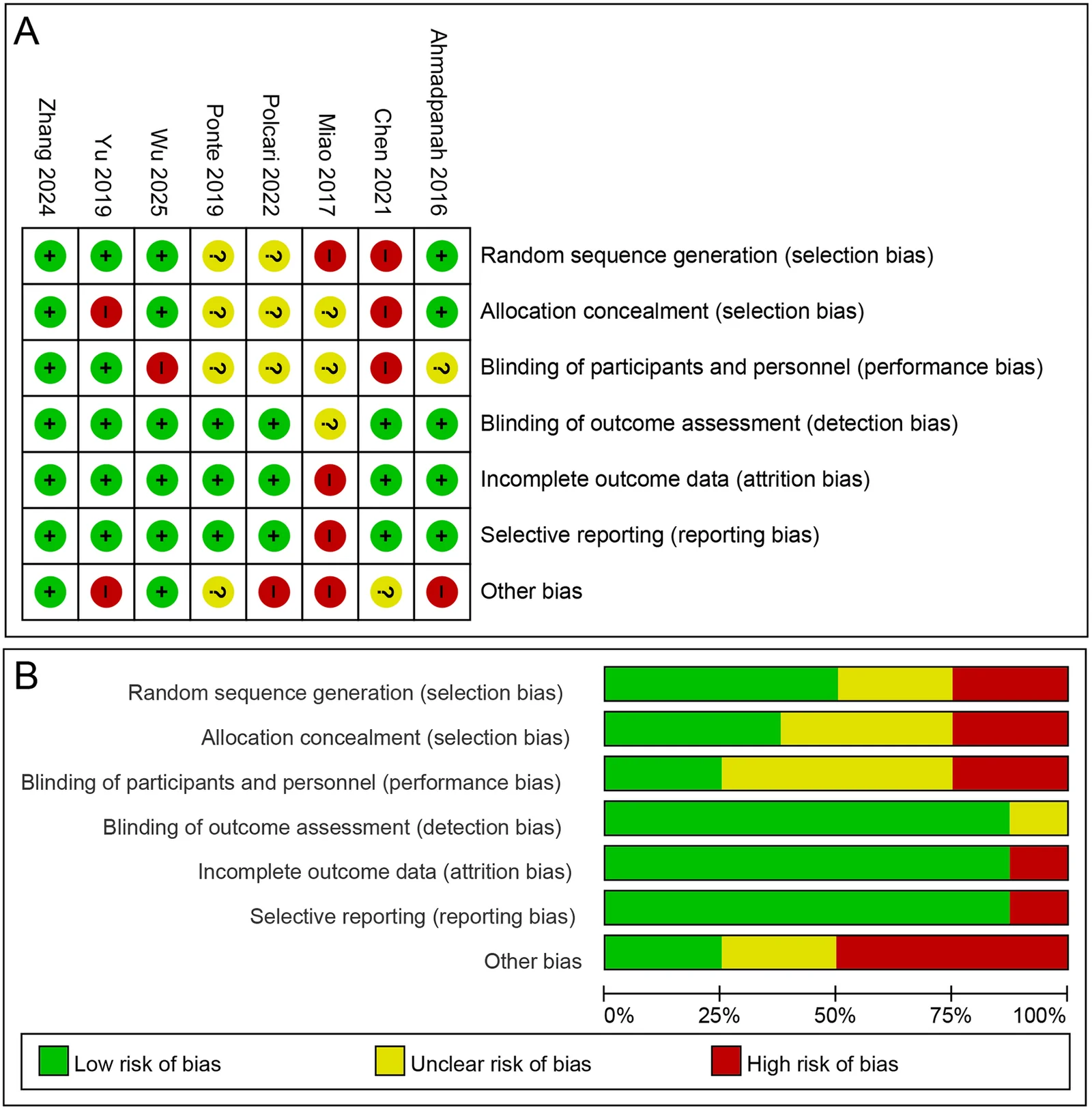
Risk of bias assessment. **(A)** Individual study risk across 7 domains; **(B)** Overall proportion per domain. Green=low, yellow=unclear, red=high risk.

### 4.3 Depression

Seven studies assessed the effects of mindfulness-based interventions on depressive symptoms. Significant heterogeneity was observed (I^2^ = 85.6%, *τ*^2^ = 0.4614, *P* < 0.0001), so a random-effects model was used. The pooled results showed that the intervention group had significantly lower depression scores than the control group (SMD = −1.19, 95% CI: −1.74 to −0.64, *P* < 0.0001).

Subgroup analysis by hypertension type showed significant effects in both subgroups. In the Essential HTN subgroup (5 studies), the random-effects pooled SMD was −1.22 (95% CI: −1.95 to −0.49), with high heterogeneity (I^2^ = 86.2%, *τ*^2^ = 0.5800, *P* < 0.0001). In the Other HTN subgroup (2 studies), the random-effects pooled SMD was −1.12 (95% CI: −2.17 to −0.06), also with high heterogeneity (I^2^ = 91.6%, *τ*^2^ = 0.5354, *P* = 0.0005). The test for subgroup differences was not statistically significant (random effects: *χ*^2^₁ = 0.03, *P* = 0.8692; common effect: *χ*^2^₁ = 0.76, *P* = 0.3832), indicating that the effect of mindfulness-based interventions on depressive symptoms was significant in both subgroups, with no statistically significant difference between essential hypertension and other hypertension (Figure 3).

**Figure 3:**
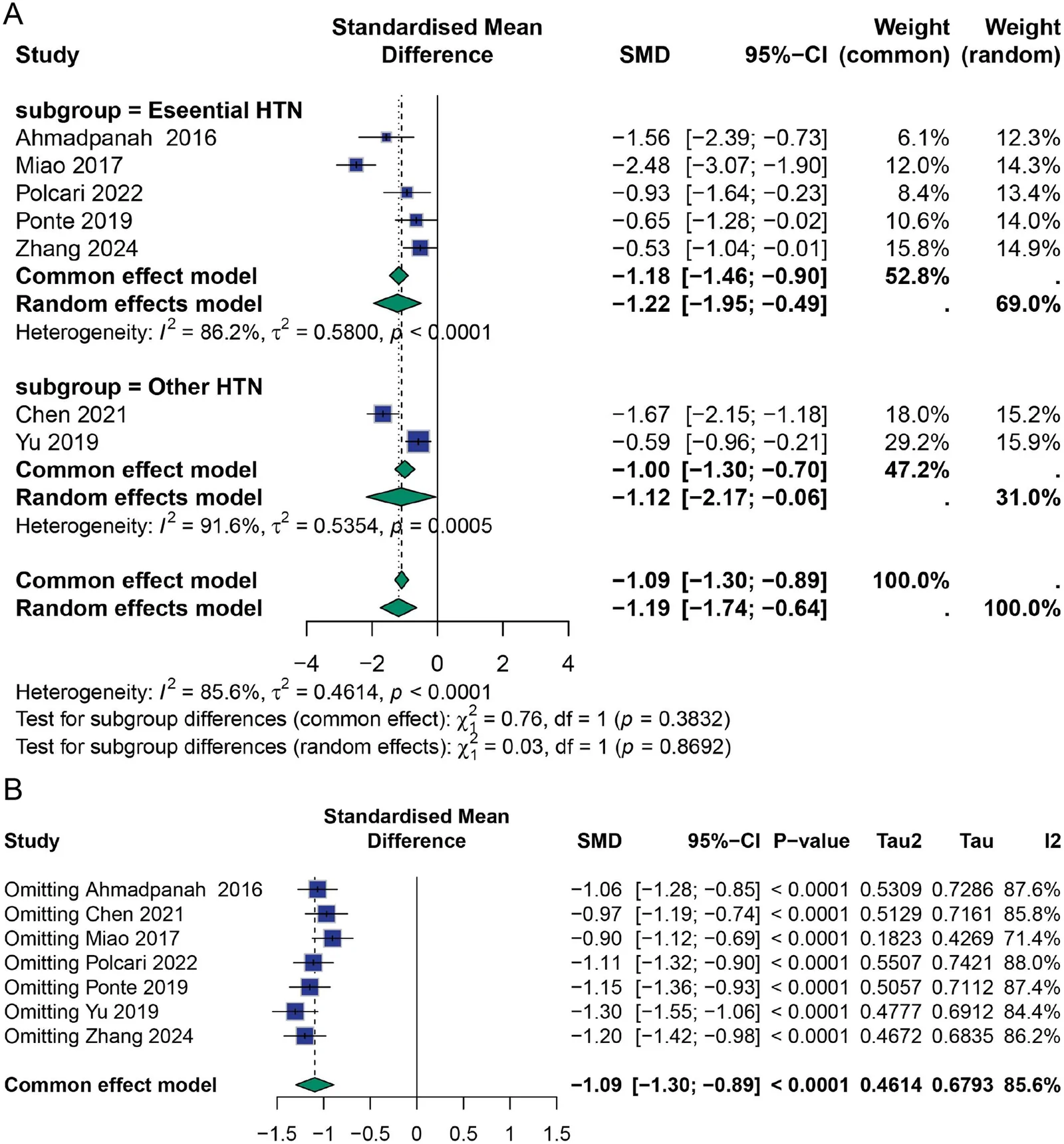
Meta-analysis and sensitivity analysis for depression. **(A)** Forest plot of SMDs; heterogeneity: I^2^ = 85.6%. **(B)** Leave-one-out sensitivity analysis showing stability of pooled effect. HTN, hypertension.

### 4.4 Anxiety

Seven studies assessed the effects of mindfulness interventions on anxiety symptoms. Considerable heterogeneity was detected (I^2^ = 94.7%, *τ*^2^ = 1.8898, *P* < 0.0001), and a random-effects model was applied. The pooled analysis showed a significant reduction in anxiety in the intervention group compared with the control group (SMD = −1.43, 95% CI: −2.48 to −0.39, *P* < 0.0001).

Subgroup analysis by hypertension type showed a significant effect in the Essential HTN subgroup (5 studies; random-effects SMD = −1.11, 95% CI: −2.12 to −0.10; I^2^ = 92.2%, *τ*^2^ = 1.2112, *P* < 0.0001). In the Other HTN subgroup (2 studies), the random-effects pooled SMD was −2.25 (95% CI: −5.33–0.83), with considerable heterogeneity (I^2^ = 98.3%, *τ*^2^ = 4.8461, *P* < 0.0001), indicating uncertainty in this subgroup estimate. The test for subgroup differences was not statistically significant (random effects: *χ*^2^₁ = 0.48, *P* = 0.4893; common effect: *χ*^2^₁ = 3.32, *P* = 0.0684), suggesting that the intervention effect on anxiety was significant in the Essential HTN subgroup, while the Other HTN subgroup estimate was uncertain, with no statistically significant difference between the two subgroups (Figure 4).

**Figure 4:**
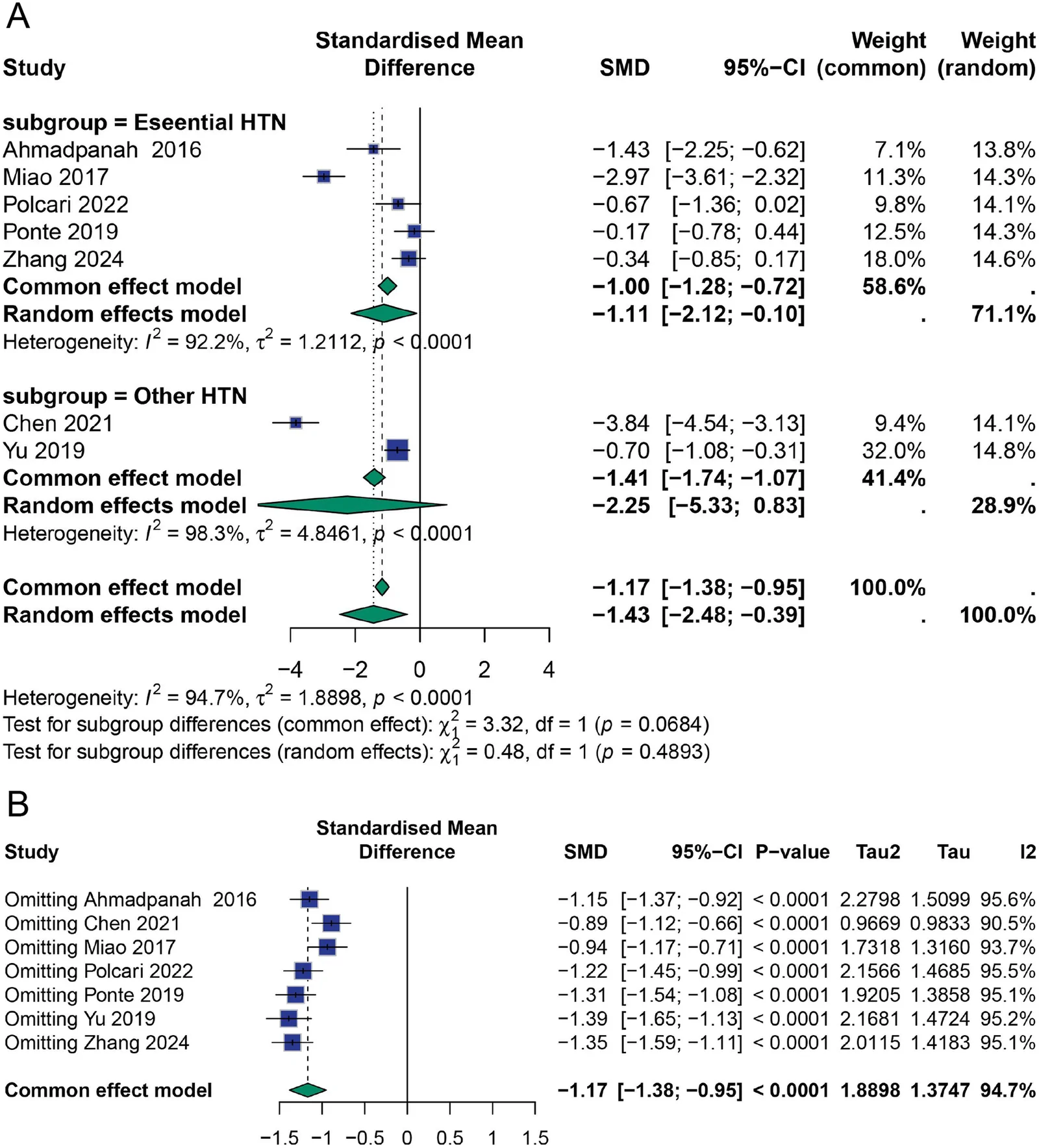
Meta-analysis and sensitivity analysis for anxiety. **(A)** Forest plot of SMDs; heterogeneity: I^2^ = 94.7%. **(B)** Leave-one-out sensitivity analysis confirming robustness of pooled effect. HTN, hypertension.

### 4.5 Perceived stress

Three studies evaluated the effects of mindfulness interventions on perceived stress. No significant heterogeneity was observed (I^2^ = 0.0%, *τ*^2^ = 0, *P* = 0.8379). Both fixed- and random-effects models yielded consistent results, showing a significant reduction in perceived stress in the intervention group (SMD = −0.53, 95% CI: −0.82 to −0.24, *P* = 0.0004). However, sensitivity analysis indicated that the result was not statistically significant after excluding the study by Yu et al. (18), which had the largest weight (59.3%) (SMD = −0.43, 95% CI: −0.88–0.03, *P* = 0.0668), suggesting that the evidence for this outcome was less stable (Figure 5).

**Figure 5:**
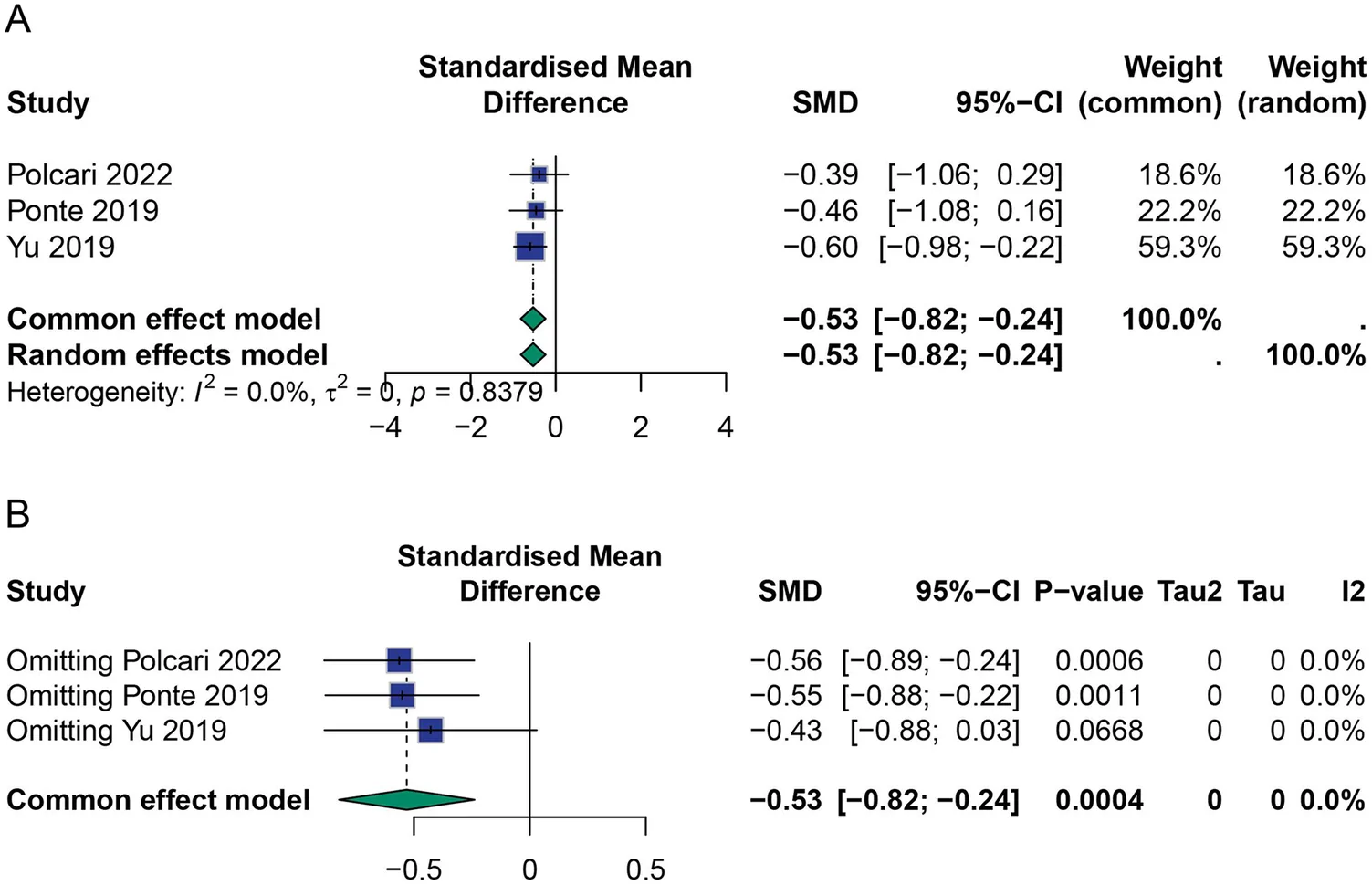
Meta-analysis and sensitivity analysis for perceived stress (PSS). **(A)** Forest plot of SMDs; no heterogeneity (I^2^ = 0%). **(B)** Leave-one-out sensitivity analysis showing consistent effect direction.

### 4.6 Blood pressure

Five studies evaluated the effects of mindfulness interventions on systolic blood pressure (SBP) and diastolic blood pressure (DBP). For SBP, substantial heterogeneity was observed (I^2^ = 93.7%, *τ*^2^ = 3.6290, *P* < 0.0001), and a random-effects model was applied. The pooled analysis showed a significant reduction in SBP in the intervention group compared with the control group (SMD = −2.10, 95% CI: −3.81 to −0.38). Leave-one-out sensitivity analysis showed that the direction and statistical significance of the effect remained unchanged, although the magnitude of the pooled effect varied, indicating that some individual studies contributed to the effect size.

Subgroup analysis based on intervention duration showed that heterogeneity remained substantial in both duration subgroups (≤8 weeks: I^2^ = 94.0%; >8 weeks: I^2^ = 95.3%). Under the random-effects model, the reduction in SBP remained significant in the >8 weeks subgroup (SMD = −2.25, 95% CI: −4.40 to −0.11), whereas the effect in the ≤8 weeks subgroup did not reach statistical significance (SMD = −2.04, 95% CI: −4.99–0.92). No significant subgroup difference was observed under the random-effects model, suggesting that intervention duration did not adequately explain the heterogeneity (Figure 6).

**Figure 6:**
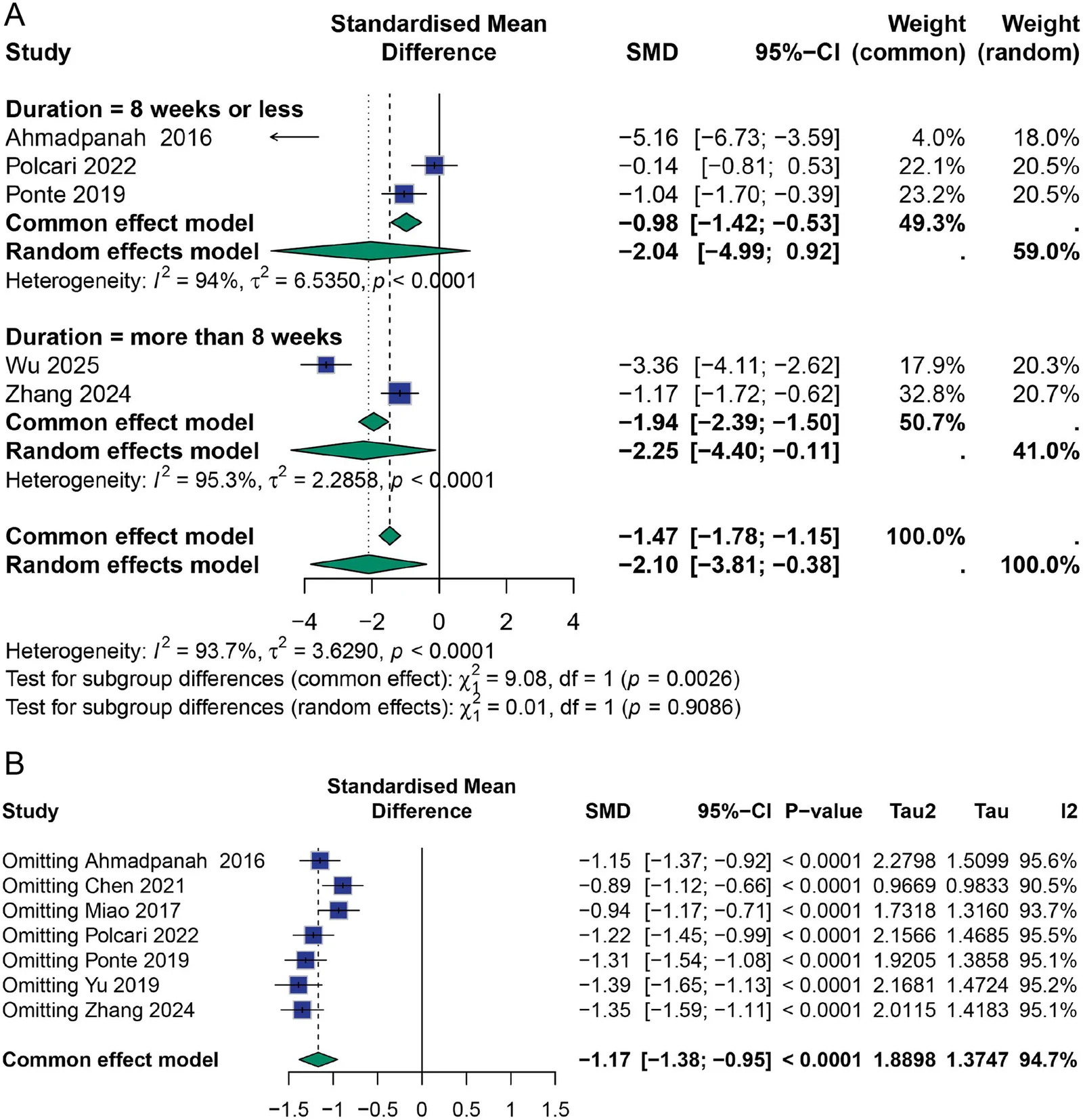
Meta-analysis and sensitivity analysis for systolic blood pressure (SBP). **(A)** Forest plot of SMDs; heterogeneity: I^2^ = 93.7%. **(B)** Leave-one-out sensitivity analysis confirming robustness despite high heterogeneity.

For DBP, substantial heterogeneity was also observed (I^2^ = 92.1%, *τ*^2^ = 1.5282, *P* < 0.0001), and the random-effects model demonstrated a significant reduction in the intervention group (SMD = −1.55, 95% CI: −2.69 to −0.42). Sensitivity analysis showed that the overall effect remained stable after excluding individual studies.

In the subgroup analysis according to intervention duration, heterogeneity remained high in both the ≤8 weeks subgroup (I^2^ = 87.1%) and the >8 weeks subgroup (I^2^ = 97.1%). Under the random-effects model, the reduction in DBP was significant in the ≤8 weeks subgroup (SMD = −1.72, 95% CI: −3.21 to −0.22), but not in the >8 weeks subgroup (SMD = −1.34, 95% CI: −3.74–1.06). The subgroup difference was not statistically significant, indicating that intervention duration alone could not explain the observed heterogeneity (Figure 7).

**Figure 7:**
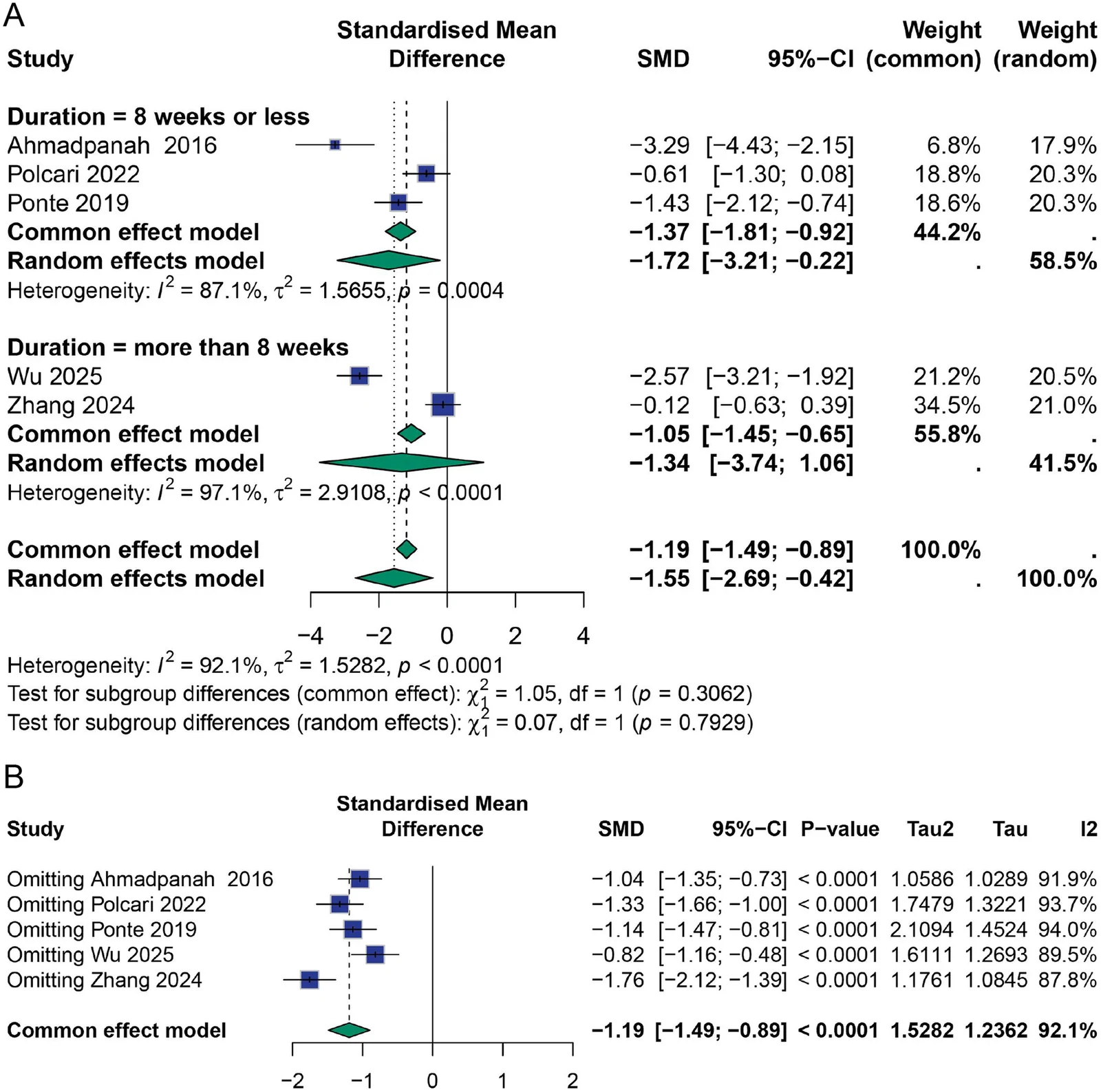
Meta-analysis and sensitivity analysis for diastolic blood pressure (DBP). **(A)** Forest plot of SMDs; heterogeneity: I^2^ = 92.1%. **(B)** Leave-one-out sensitivity analysis showing consistent effect direction despite high heterogeneity.

### 4.7 GRADE rating

The certainty of evidence for each outcome was evaluated using the GRADE approach (Table 2). The certainty of evidence for improvements in depressive symptoms and reductions in systolic and diastolic blood pressure was rated as moderate. This rating was primarily downgraded due to serious risk of bias and significant inconsistency observed in these outcomes. In contrast, the certainty of evidence for anxiety, perceived stress, and quality of life was rated as low. Beyond the aforementioned limitations regarding risk of bias and inconsistency, the evidence for anxiety and quality of life was further downgraded because most trials had unclear methodological reporting. Furthermore, the outcomes of perceived stress and quality of life were downgraded for serious imprecision due to the small number of included trials and limited sample sizes, which compromised the stability of the pooled estimates. Although the meta-analysis suggests positive effects of mindfulness-based interventions on psychological well-being and blood pressure control, these findings should be interpreted with caution given the methodological limitations and inconsistency of the available evidence. Future large-scale, rigorously designed randomized controlled trials with longer follow-up periods are needed to verify the robustness and long-term sustainability of these effects.

**Table 2:** Evidence profile for each outcome.

No. of studies	Outcomes	Assessment	SMD	95% CI	Risk of bias	Inconsistency	Indirectness	Imprecision	Other consideration	Certainty
7	Depressive symptoms	Various scales	−1.19	−1.74, −0.64	Serious^a^	Serious^b^	Not serious	Not serious	None	⊕⊕◯◯ Moderate^a^^,^^b^
7	Anxiety	Various scales	−1.43	−2.48, −0.39	Serious^c^	Serious	Not serious	Not serious	None	⊕◯◯◯ Low^a^^,^^c^
3	Perceived stress decreased	Perceived Stress Scale	−0.53	−0.82, −0.24	Serious^a^	Not serious	Not serious	Seriousᵈ	None	⊕◯◯◯ Low^a^^,^^d^
5	Systolic Blood Pressure	SMD	−2.10	−3.81, −0.38	Serious^a^	Serious^b^	Not serious	Not serious	None	⊕⊕◯◯ Moderate^a^^,^^c^
5	Diastolic Blood Pressure	SMD	−1.55	−2.69, −0.42	Serious^a^	Serious^b^	Not serious	Not serious	None	⊕⊕◯◯ Moderate^a^^,^^c^

CI, confidence interval; SMD, standardized mean difference; SDS, self-rating depression scale; SAS, self-rating anxiety scale.

Explanations.

a Unclear allocation concealment and blinding.

b Significant heterogeneity.

c Most studies had unclear methodological details.

d Small-sample size.

## 5 Discussion

Hypertension is a chronic condition that often coexists with persistent psychological distress (20). Anxiety, depressive symptoms, and heightened perceived stress are not only common among patients with elevated blood pressure but may also interfere with long-term self-management and treatment adherence. In recent years, mindfulness-based interventions have been increasingly introduced into chronic disease management as a potential strategy to address emotional burden alongside physiological control (21). Against this background, the present meta-analysis synthesized available randomized evidence to clarify their role in hypertensive populations (22). Overall, our findings suggest that mindfulness-based interventions were associated with improvements in negative emotional outcomes and produced modest reductions in blood pressure compared with control conditions.

The psychological improvements observed in this review represent the most consistent finding. Across studies, participants receiving mindfulness-based interventions experienced reductions in depressive symptoms, anxiety, and perceived stress, suggesting that these programs may effectively address the emotional burden commonly associated with hypertension. Beyond general emotional regulation, mindfulness training may help patients regulate maladaptive cognitive patterns, such as rumination, disease-related worry, and heightened symptom vigilance, thereby potentially reducing psychological burden and improving coping with long-term treatment requirements (23). For individuals living with a lifelong condition that often requires sustained medication use and lifestyle modification, improved emotional stability may also enhance self-management behaviors, including treatment adherence, health monitoring, and engagement in lifestyle changes. In this context, the psychological benefits observed in this analysis are likely to have practical clinical relevance, even when the intervention is not delivered as a formal mental health therapy (24).

In contrast, the effects on blood pressure were present but less pronounced, suggesting that the effects of mindfulness on cardiovascular outcomes may involve indirect and multifactorial pathways. Psychological distress and sustained stress responses are known to contribute to sympathetic activation, behavioral dysregulation, and poor treatment adherence, all of which can adversely affect blood pressure control (25). By reducing emotional reactivity and improving stress coping, mindfulness may help stabilize daily blood pressure fluctuations rather than producing large absolute reductions. The modest magnitude observed in the pooled analysis may also reflect the clinical characteristics of the included populations, as many participants were already receiving antihypertensive treatment, potentially limiting the additional improvement achievable through behavioral intervention alone (26). Furthermore, differences in baseline blood pressure levels, intervention intensity, duration, and concomitant treatment may have contributed to variability in the observed effects, indicating that longer-term and more standardized interventions may be needed to clarify the cardiovascular benefits of mindfulness-based interventions.

These findings should be interpreted with caution in light of several limitations. Although significant effects were observed, substantial heterogeneity existed across several outcomes, which may reflect differences in intervention intensity, duration, participant characteristics, and outcome assessment among studies (27, 28). Subgroup analyses based on hypertension type and intervention duration were conducted to explore potential sources of heterogeneity; however, these factors did not fully explain the observed variability, suggesting that heterogeneity may be multifactorial. In addition, the overall number of trials and total sample size were relatively limited, and some studies included small cohorts, which may affect the stability of the pooled estimates (29). Psychological outcomes were not consistently pre-specified as primary endpoints across studies, which may have contributed to methodological heterogeneity. Randomization procedures were not clearly reported in some studies, leading to uncertainty in the risk of selection bias. Because mindfulness is a behavioral intervention, participant blinding was generally not feasible, potentially influencing self-reported psychological outcomes (30). Moreover, information regarding antihypertensive medication regimens and the use of antidepressant or anxiolytic medications was incompletely reported, limiting further adjustment or subgroup analyses based on medication-related factors. Furthermore, most trials evaluated short-term effects only, and the long-term sustainability of both psychological and blood pressure improvements remains uncertain. The evidence regarding quality of life was also limited, as only a small number of studies reported this outcome, preventing a robust quantitative synthesis. Future trials should incorporate standardized quality-of-life assessments and longer follow-up periods to better determine the comprehensive benefits of mindfulness interventions in patients with hypertension.

Overall, mindfulness-based interventions appear to provide meaningful improvements in psychological well-being among individuals with hypertension and may contribute to modest support for blood pressure control (31). Rather than replacing pharmacological therapy, mindfulness may be considered a complementary strategy within comprehensive hypertension management. Future large-scale, well-designed randomized trials with standardized intervention protocols and longer follow-up periods are needed to clarify the durability and clinical significance of these effects.

## Data Availability

The original contributions presented in the study are included in the article/Supplementary Material, further inquiries can be directed to the corresponding authors.
