# Supplementary material for: Effects of mindfulness-based interventions on negative emotions in patients with hypertension: a systematic review and meta-analysis
Source: Front Cardiovasc Med. 2026 Oct 30;13:1941304. doi: 10.3389/fcvm.2026.1941304 (PMC13759840; doi:10.3389/fcvm.2026.1941304)
Supplement: Supplementary file 1 [file Table1.docx]

**Supplementary Table 1. Search strategies for each database**

| Database | Search Strategy |
| --- | --- |
| PubMed | (Hypertension[Mesh] OR hypertension OR "high blood pressure" OR "elevated blood pressure") AND (Mindfulness[Mesh] OR mindfulness OR "mindfulness-based" OR "mindfulness-based stress reduction" OR MBSR OR "mindfulness-based cognitive therapy" OR MBCT OR meditation) AND (Anxiety[Mesh] OR Depression[Mesh] OR "Stress, Psychological"[Mesh] OR "Quality of Life"[Mesh] OR anxiety OR depression OR stress OR "negative emotion*" OR "emotional distress" OR "quality of life" OR QoL OR HRQoL) AND (randomized controlled trial OR randomized OR randomised OR RCT) |
| Embase | ('hypertension'/exp OR hypertension OR 'high blood pressure') AND ('mindfulness'/exp OR mindfulness OR 'mindfulness based' OR 'mindfulness based stress reduction' OR mbsr OR 'mindfulness based cognitive therapy' OR mbct OR meditation) AND ('anxiety'/exp OR 'depression'/exp OR 'psychological stress'/exp OR 'quality of life'/exp OR anxiety OR depression OR stress OR 'negative emotion*' OR 'emotional distress' OR 'quality of life' OR qol) AND ('randomized controlled trial'/exp OR random* OR rct) |
| Cochrane Library | (hypertension OR "high blood pressure") AND (mindfulness OR "mindfulness-based" OR "mindfulness-based stress reduction" OR MBSR OR "mindfulness-based cognitive therapy" OR MBCT OR meditation) AND (anxiety OR depression OR stress OR "negative emotion*" OR "emotional distress" OR "quality of life" OR QoL) |
| Web of Science | TS = (hypertension OR "high blood pressure") AND TS = (mindfulness OR "mindfulness-based" OR "mindfulness-based stress reduction" OR MBSR OR "mindfulness-based cognitive therapy" OR MBCT OR meditation) AND TS = (anxiety OR depression OR stress OR "negative emotion*" OR "emotional distress" OR "quality of life" OR QoL OR HRQoL) |
| CNKI | SU=(hypertension) * (mindfulness OR mindfulness intervention OR mindfulness-based stress reduction OR mindfulness-based cognitive therapy OR mindfulness therapy OR meditation) * (anxiety OR depression OR negative emotions OR psychological stress OR emotional disorders OR quality of life OR health-related quality of life) |
