# Supplementary material for: Effects of mindfulness-based interventions on negative emotions in patients with hypertension: a systematic review and meta-analysis
Source: Front Cardiovasc Med. 2026 Oct 30;13:1941304. doi: 10.3389/fcvm.2026.1941304 (PMC13759840; doi:10.3389/fcvm.2026.1941304)
Supplement: Supplementary file 2 [file Table2.docx]

**Supplementary Table 2. Characteristics of the included studies, hypertension populations, psychological assessment methods, antihypertensive medication use, and mindfulness-based interventions**

| First author | Publication year | Hypertention population | Mindfulness intervention | Psychological assessment tools | Antihypertensive medication |
| --- | --- | --- | --- | --- | --- |
|  |  |  |  |  |  |
| Ahmadpanah^[13]^ | 2016 | Women with hypertension | Duration: 8 weeks Format: Weekly 1-hour group sessions Core Content: - MDM: Attention training techniques, metacognitive exercises, imagery training (e.g., "clouds and train station" exercise). - SMT: Progressive muscle relaxation, systematic desensitization, cognitive restructuring, diaphragm breathing, and self-creation training. | Depression: Beck Depression Inventory (BDI) Anxiety: Beck Anxiety Inventory (BAI) | All patients continued their individual antihypertensive medication regimens, which were kept constant throughout the study period until the follow-up. |
| Chen^[14]^ | 2021 | Pregnant women with gestational hypertension | Duration: In-hospital phase (1–2 weeks) + 1-month home practice. Format: Daily in-hospital guidance by trained nurses + self-practice with manuals/videos and WeChat group support for check-ins. Core Content: Body scan, mindful breathing, and mindfulness meditation. | Depression: Hamilton Depression Scale (HAMD) Anxiety: Hamilton Anxiety Scale (HAMA) | NR |
| Miao^[15]^ | 2017 | Hypertensive patients with comorbid anxiety and depression. | Duration: 1 month Format: Daily sessions (≥30 minutes each), guided by researchers. Core Content: Mindful breathing, body scanning, and daily life meditation training (e.g., being aware of routine activities like brushing teeth, washing face). | Anxiety: Self-Rating Anxiety Scale (SAS) Depression: Self-Rating Depression Scale (SDS) | NR |
| Polcari^[16]^ | 2022 | Participants with elevated blood pressure or hypertension | Duration: 8 weeks Format: Weekly 2.5-hour group sessions + one 7.5-hour retreat day. Core Content: Standard MBSR curriculum (body scan, meditation, yoga) plus education on hypertension risk factors (diet, physical activity, alcohol, medication adherence) and modules applying mindfulness skills specifically to these determinants of blood pressure. | Depression: Center for Epidemiologic Studies Depression Scale Revised (CESD-R) Anxiety: Beck Anxiety Inventory (BAI) Other: Perceived Stress Scale (PSS-10) | Participants continued their usual antihypertensive treatment. Medication use was monitored but remained stable during the study. |
| Ponte^[17]^ | 2019 | Mediterranean patients with high-normal blood pressure or Grade I hypertension | Duration: 8 weeks Format: Weekly 2-hour group sessions. Core Content: Mindfulness of breath, bodily sensations, thoughts, sounds, and everyday activities. Patients were encouraged to practice at home for 45 minutes daily. | Perceived Stress: Perceived Stress Scale (PSS-10) | 65% of patients were on antihypertensive treatment. Medication regimens and doses were maintained unchanged for both groups throughout the study. |
| Wu^[18]^ | 2025 | Patients with intradialytic hypertension (IDH) undergoing maintenance haemodialysis | Duration: 12 weeks Format: Guided audio session (62 minutes) delivered during each dialysis session, with noise-cancelling headphones and a quiet environment. Core Content: Body scan, breath awareness, visualisation (imagining blood being cleansed by the dialysis machine), healing sound waves (music), and stress release (sensory exploration). | NR | All patients received standardised antihypertensive treatment and erythropoiesis-stimulating agents (ESAs), which were consistently maintained throughout the study. |
| Yu^[19]^ | 2019 | Pregnant women with gestational hypertension | Duration: 8 weeks Format: Weekly 2–3 sessions (≥1 hour each), combining in-hospital guidance and daily home practice. Core Content: Body scanning, mindful breathing, mindfulness meditation, mindful walking, and mindful yoga. | Depression: Hamilton Depression Scale (HAMD) Anxiety: Hamilton Anxiety Scale (HAMA) Perceived Stress: Perceived Stress Scale (PSS) | NR |
| Zhang^[20]^ | 2024 | Community-dwelling hypertensive patients | Duration: 10 weeks Format: Weekly education sessions from specialised psychologists + Tai Chi sessions with relaxation music. Core Content: Relaxation training, negative thinking recognition, cognitive behaviour improvement, broadening mental flexibility, mindfulness exercises, communication skills, plus standard hypertension health education. | Depression: Patient Health Questionnaire (PHQ-9) Anxiety: Generalized Anxiety Disorder Scale (GAD-7) Self-Efficacy: Self-efficacy scale (6 items) | All participants were hypertensive patients under community management, receiving standard antihypertensive treatment. Patients taking antipsychotic or anti-anxiety/depression medications in the past 3 months were excluded. |
